# Effects of music therapy and music-based interventions in the treatment of substance use disorders: A systematic review

**DOI:** 10.1371/journal.pone.0187363

**Published:** 2017-11-15

**Authors:** Louisa Hohmann, Joke Bradt, Thomas Stegemann, Stefan Koelsch

**Affiliations:** 1 Department for Educational Sciences and Psychology, Freie Universität, Berlin, Germany; 2 Department for Biological and Medical Psychology, University of Bergen, Bergen, Norway; 3 Department of Creative Arts Therapies, College of Nursing and Health Professions, Drexel University, Philadelphia, United States of America; 4 Department of Music Therapy, University of Music and Performing Arts, Vienna, Austria; Stanford University School of Medicine, UNITED STATES

## Abstract

Music therapy (MT) and music-based interventions (MBIs) are increasingly used for the treatment of substance use disorders (SUD). Previous reviews on the efficacy of MT emphasized the dearth of research evidence for this topic, although various positive effects were identified. Therefore, we conducted a systematic search on published articles examining effects of music, MT and MBIs and found 34 quantitative and six qualitative studies. There was a clear increase in the number of randomized controlled trials (RCTs) during the past few years. We had planned for a meta-analysis, but due to the diversity of the quantitative studies, effect sizes were not computed. Beneficial effects of MT/ MBI on emotional and motivational outcomes, participation, locus of control, and perceived helpfulness were reported, but results were inconsistent across studies. Furthermore, many RCTs focused on effects of single sessions. No published longitudinal trials could be found. The analysis of the qualitative studies revealed four themes: emotional expression, group interaction, development of skills, and improvement of quality of life. Considering these issues for quantitative research, there is a need to examine social and health variables in future studies. In conclusion, due to the heterogeneity of the studies, the efficacy of MT/ MBI in SUD treatment still remains unclear.

## Introduction

The misuse of legal and illegal substances is a significant problem in modern societies. For example, in the United States, the estimated 12-months prevalence rates for addictions in 2014 were 3.0% for alcohol and 1.9% for illicit drugs [1]. Use and misuse of alcohol and drugs are associated with a variety of health, social, and economic disadvantages for the users themselves and others (e.g., family, friends, community, environment, and country [2]). Treatment programs for patients with substance use disorders (SUD) include body detoxification, pharmaceutical, psychosocial, and psychotherapeutic treatment, and recovery management [3]. Nevertheless, only a minority of people with SUD, i.e., about 10%, receives such professional help [4]. Moreover, the treatment completion rates are low (i.e., 47% in the USA in 2006 [5]) and the relapse rates are high (40–60% [6]). Thus, there is still need to improve addiction treatment.

Standard psychological treatments mostly consist of verbal therapies such as cognitive behavior therapy, motivational interviewing, and relapse prevention [7]. In addition, complementary and alternative medical therapies are utilized to allow for creative and expressive ways to address issues. Music therapy is one of such non-mainstream therapies [8]. According to the American Music Therapy Association [9], music therapy is defined as the “clinical and evidence-based use of music interventions to accomplish individualized goals within a therapeutic relationship by a credentialed professional who has completed an approved music therapy program”. Therefore, in this review, the term *music therapy* (MT) is used only for studies where music therapists were involved in the delivery of the intervention; for studies where the intervention was delivered without participation of music therapists, or their participation remains unclear, we will use the term *music-based intervention* (MBI). Furthermore, we include studies examining the effect of music stimuli presentation without presence of persons therapeutically guiding the interventions, which are referred to as *music presentation* (MP) studies.

How can MT/ MBI help patients with SUD? Compared to commonly used verbal psychological therapies, MT and MBI provide different opportunities for self-expression, cooperative group activity, imagination, and synchronized sensorimotor experience [10]. In addition to that, there is evidence of beneficial impact of MT/ MBI on mood [11,12], stress [13], self-esteem [14], motivation [15], emotional expression [16], and social cohesion [17]. Furthermore, MT/ MBIs appear to address general challenges of SUD treatment: For instance, in a study with patients with SUD and comorbid severe mental illnesses MT appreciation was associated with benefits in global functioning and motivation [15]. For patients with non-organic mental disorders and low treatment motivation positive effects of an individual three month MT program on negative symptoms, global functioning, clinical global impressions, social avoidance and vitality were reported [18]. Furthermore, for subgroups of addicted patients with special needs (e.g., women and adolescents [8]) MT/ MBI led to improvements in anxiety [19] and internal locus of control [20].

To clarify the clinical efficacy of MT/ MBIs in addiction treatment, a summary of their effects is warranted. Although there are many reports about the effects of MT/ MBI in patients with SUD in single studies, no meta-analyses are yet available on this topic. In 2008, Mays, Clark, and Gordon [21] systematically reviewed the use of MT for patients with SUD and emphasized a lack of evidence. In their review, they included five quantitative studies that greatly varied in terms of treatment settings, frequency, duration, persons guiding the session, and outcome variables. Furthermore, outcomes like drug consumption or long-term abstinence were not assessed in these studies. Therefore, the treatment effects of MT were primarily related to participants’ attitudes and emotions. In line with that, most of the MT studies in SUD treatment met the criteria of lower levels of evidence according to evidence-based practice hierarchies, indicating that high-quality research has not been conducted [22].

In this paper, we aimed to address the research question of whether MT and MBIs are clinically effective for people with substance use disorders (SUD) by reviewing the current state of research regarding this topic. Because little is known about the key outcomes affected by MT/ MBIs in patients with SUD [21], we evaluated the existing evidence to summarize the benefits of music interventions for this population.

## Methods

### Criteria for considering studies for this review

#### Types of studies

We included all types of studies with quantitative or qualitative data assessed in a systematic way, e.g., by at least semi-structured interviews, video-taping, or questionnaires. We decided not to limit our inclusion criteria to randomized controlled trials (RCTs), even though there are many scholars who recommend focusing on this type of study for systematic reviews and meta-analyses [22,23]. We based this decision on the following rationale: (1) Silverman [22] and Mays et al. [21] emphasized the lack of RCTs available for our research question, and this is still valid at present; (2) for rare conditions and difficult clinical investigations (such as music therapy in psychiatry) the inclusion of other study types (such as case studies or case-control studies) is recommended because they may be the only available evidence [24]; (3) Furthermore, qualitative studies are useful to examine perspectives and experiences [22,25].

We also included MP studies examining the effects of music stimuli presentation on people with SUD without the presence of a music therapist or other persons therapeutically guiding the music intervention.

#### Types of participants

We considered studies that included patients or clients with SUD, regardless of age, gender or comorbid disorders. Studies examining subgroups like women or adolescents were included as well. If it was unclear whether all participants suffered from SUD (e.g., a study on residents and staff members of a rehabilitation center [26]), those studies were excluded. If separate conclusions about patients with and without SUD were drawn, those studies were included.

#### Types of interventions

All studies examining MT, MBI or MP were included. Articles were excluded if combined programs with music and other complementary approaches were used (e.g., combinations of art, video, music, group therapy, and individual counseling [27]) as this would not allow for the identification of separate effects of MT/ MBI/ MP.

#### Types of outcome measures

Similar to Mays et al. [21], we included all outcomes. For a listing of the outcomes included in the study, see Table 1.

**Table 1:** Clusters of outcomes examined in studies about the effects of music therapy and music-based interventions for patients with substance use disorders.

Outcome label	Included variables	Studies
Motivation	Treatment eagerness	Silverman [29,34,36]
	Change readiness/ Readiness to change	Silverman [31,32]
	Motivation	Silverman [32,33,35], K. M. Murphy [38], Ross et al. [15], Baker et al. [16]
	Motivation for sobriety	Silverman [34]
	Motivation to reach and maintain sobriety	Silverman [36]
Depression	Depression	Albornoz [39], K. M. Murphy [38], Oklan & Henderson [40], Silverman [31], Yun & Gallant [41]
	Depressiogenic thought frequency	Howard [42]
	Feeling depressed	Cevasco et al. [43], Gallant et al. [44], Hwang & Oh [45], Jones [46]
Enjoyment	Perceived enjoyment	Baker et al. [16], Silverman [29,31,32,47]
	Feeling of joy/happiness/enjoyment	Jones [46]
Withdrawal/ craving	Withdrawal symptoms	Silverman [30,37]
	Craving	Silverman [32,37]
Helpfulness	Perceived helpfulness	Gallant et al. [44], Silverman [31,32]
	Music therapy appreciation	Ross et al. [15]
	Perceived therapeutic effectiveness	Silverman [47]
Locus of control	Locus of control	James [20], Silverman [30]
Participation	Working alliance	Silverman [29]
	Treatment retention and completion	Dickerson et al. [48]
	Adoption of the program	Dickerson et al. [48]
	Active participation	Gallagher & Steele [49]
	Sociability	Gallagher & Steele [49]
	Participation in the processing session	Gallagher & Steele [49]
	Attendance	Dougherty [50], Baker et al. [16], Ross et al. [15]
Coping skills	Coping skills	K. M. Murphy [38], Oklan & Henderson [40]
	Knowledge of triggers and coping skills	Silverman [36]
Anxiety	Psychiatric symptom	Ross et al. [15]
	Emotional experience	Cevasco et al. [43], Gallant et al. [44], Gardstrom & Diestelkamp [19], Gardstrom et al. [51], Hwang & Oh [45], Jones [46]
	Trait	Cevasco et al. [43]
Medical symptoms	General functioning	Dickerson et al. [48], Ross et al. [15]
	Physical symptoms	Dickerson et al. [48]
	Psychiatric symptoms	Oklan & Henderson [40]
Anger	Emotional experience	Cevasco et al. [43], Gardstrom et al. [51], Hwang & Oh [45]
Sadness	Feeling sad	Gardstrom et al. [51]
	Feeling unhappy	Gallant et al. [44]
Stress		Cevasco et al. [43], Hwang & Oh [45]

### Search methods for identification of studies

First, we identified articles by conducting a literature search in the electronic databases *ISI Web of Knowledge* and *Scopus* on 1^st^ April, 2016. We used the search term “(music therapy AND addiction) OR (music therapy AND substance use disorder) OR (music therapy AND substance abuse) OR (music therapy AND alcohol*) OR (music AND intervention AND addiction)) OR (music AND intervention AND substance use disorder) OR (music AND intervention AND substance abuse) OR (music AND intervention AND alcohol*)”. After deleting duplicate studies, we scanned the abstracts to include only articles published in English, focusing on MT/ MBI or MP and participants with SUD. Additionally, the bibliographies of the remaining records were scanned for further studies. Articles without systematic data assessment were excluded. Remaining sources were further subdivided with respect to the type of music/ intervention that was examined: (1) studies examining effects of the presentation of music stimuli without application of MT/ MBI (MP studies), (2) studies investigating one session of MT/ MBI, and (3) studies examining more than one session of MT/ MBI. With respect to category (1), for example examinations of simple listening to music without the presence of therapists or other persons guiding the session or experiments were included.

### Data collection and analysis

#### General preparing procedure

A review protocol does not exist. All unique articles (i.e. duplicates removed) were listed in a table. After their abstracts were scanned, we indicated whether or not the studies met the inclusion criteria listed above. Full texts of studies that met the inclusion criteria were analyzed. The study characteristics and results were summarized in separate tables.

Many studies included similar outcomes but used different terminology. Outcomes that were very similar were clustered under one common outcome term. For example, the outcomes depression, depressiogenic thought frequency, and feeling depressed were clustered under the outcome “depression” (See Table 1 for labels and included variables). For all studies, we extracted design aspects as well as statistical data. Based on this data, we examined if meta-analytic calculations would be useful.

We used three different types of data summary: (1) a description of the effects of MT/ MBI for the quantitative studies separated by outcomes, (2) a summary of effects of MT/ MBI/ MP for the quantitative studies separated by study characteristics, (3) a summary of the topics and themes described in the qualitative studies.

We did not conduct a meta-analysis due to the following reasons. First, according to the Cochrane systematic review guidelines [23], combining studies that use different types of control conditions may lead to meaningless results. After separating the studies per type of control condition, there were too few studies per outcome to allow for meta-analysis. Second, predominantly including studies by the same authors in the same meta-analysis would violate the assumption of independence of study reports [28]. As most of the studies with similar comparison designs were conducted by Silverman [29–37], there was too much dependency on the hierarchical level. A more detailed description of reasons for not conducting a meta-analysis is provided in the Results section below.

#### Descriptive summaries

**Fig 1:**
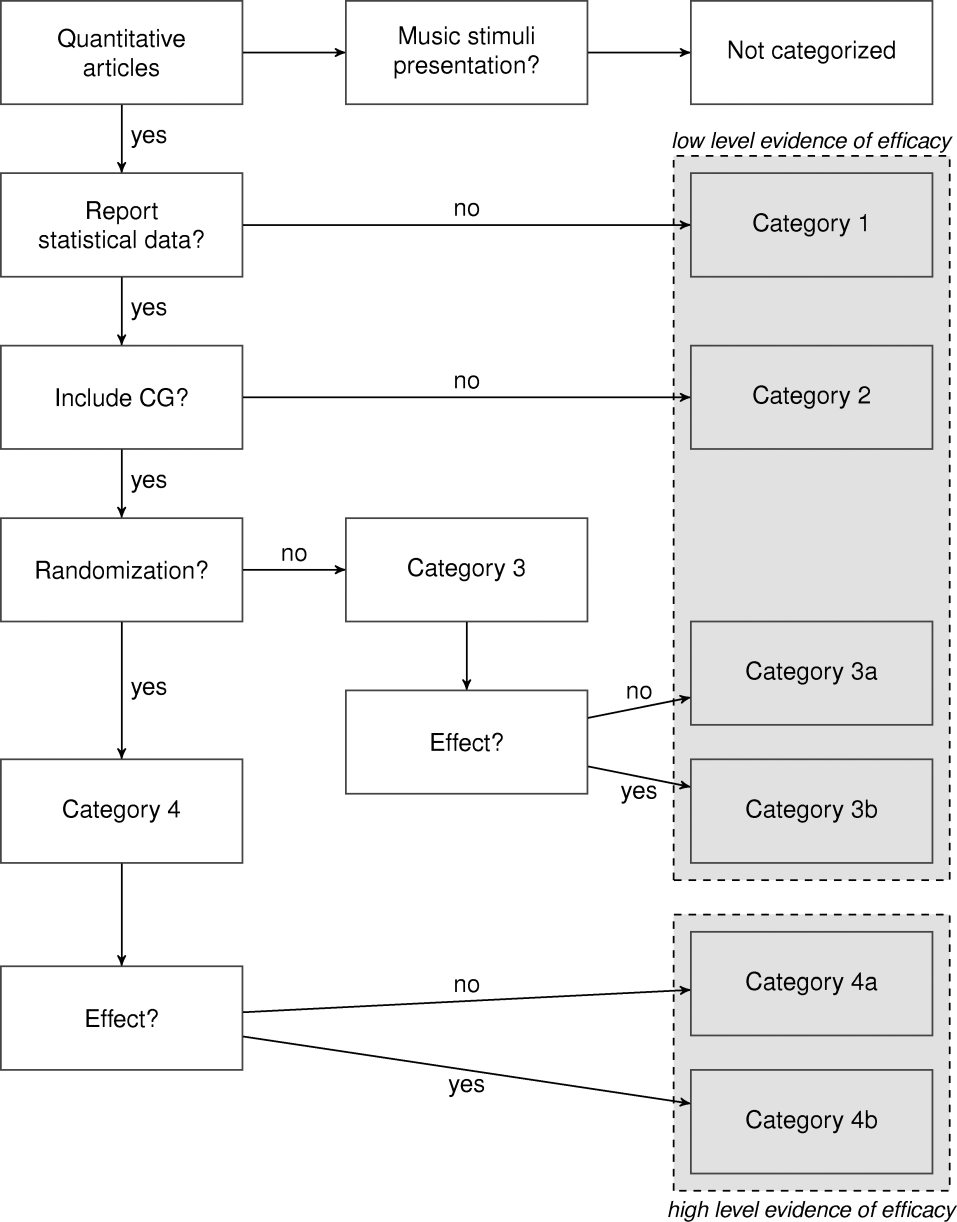
Categorization procedure for quality of evidence. CG = control group.

We aimed to give an overview of the efficacy of MT/ MBI per outcome in consideration of the quality of the studies. To this end, we created a categorization system (see Fig 1) based on an evidence-based practice (EBP) taxonomy by Melnyk and Fineout-Overholt [52] that was developed for the nursing profession. As MT and nursing contexts appear to be similarly diverse, Silverman [22] recommended the use of this taxonomy when examining EBP for MT. This hierarchy contains seven levels of evidence with (I) being the highest rank and (VII) being the lowest rank in research. The articles we collected for our review did not cover the whole range. Therefore, we refer to Melnyk and Fineout-Overholt’s following levels: (II) well-designed RCTs, (III) well-designed controlled trials without randomization, and (VI) single descriptive or qualitative study. Based on these levels, we developed four main categories for our categorization system: (1) studies without reporting all necessary statistical data to compute a meta-analysis (e.g., means, standard deviations, sample sizes), (2) studies without a control group (CG), (3) non-randomized studies with CG, and (4) RCTs. Categories (3) and (4) are further subdivided into (3a)/ (4a) studies that reported no beneficial treatment effects of MT/ MBI and (3b)/ (4b) studies that reported treatment benefits of MT/MBI compared to a CG. For an overview of the categorization procedure see Fig 1. To draw conclusions about MT/ MBI efficacy, RCTs are necessary [25]. Thus, studies fitting in categories (4a) and (4b), which are matching level (II) of Melnyk and Fineout-Overholt’s taxonomy, are categorized as *high level evidence of efficacy*. Categories (3a) and (3b) match level (III) in the EBP taxonomy, and categories (1) and (2) match level (VI), i.e., lower levels of evidence. Thus, the categories (1), (2) and (3a)/(3b) are referred to as of *low level evidence of efficacy*. Nevertheless, it is important to note that research designs other than RCTs are useful for research as well [25], so that our taxonomy of *low and high level of evidence of efficacy* only refers to the assessment of MT/ MBI efficacy.

For the descriptive summaries we used the following rules: We counted how many unique studies examined a certain outcome (cluster). For studies that included multiple measures (e.g. two different scales) per outcome, data from only one measure was included. This was to avoid artificially inflating the weight of single studies. Articles that reported results of two separate studies within a single publication were used more than once (e.g., [20]). If different raters (e.g. client ratings and therapist ratings) were included only client ratings were counted. Finally, for studies with repeated measures, only immediate post-intervention scores were used.

#### Summary of music and MT/ MBI effects

We created separate summaries for (1) MP studies, (2) studies that investigated only one session, and (3) studies that examined the effects of more than one session of MT/ MBI. For each of these three categories a separate table including study characteristics and results was created.

#### Summary of qualitative articles

Studies were read carefully, and described topics and themes were summarized in a separate table.

## Results

### Description of the studies

**Fig 2:**
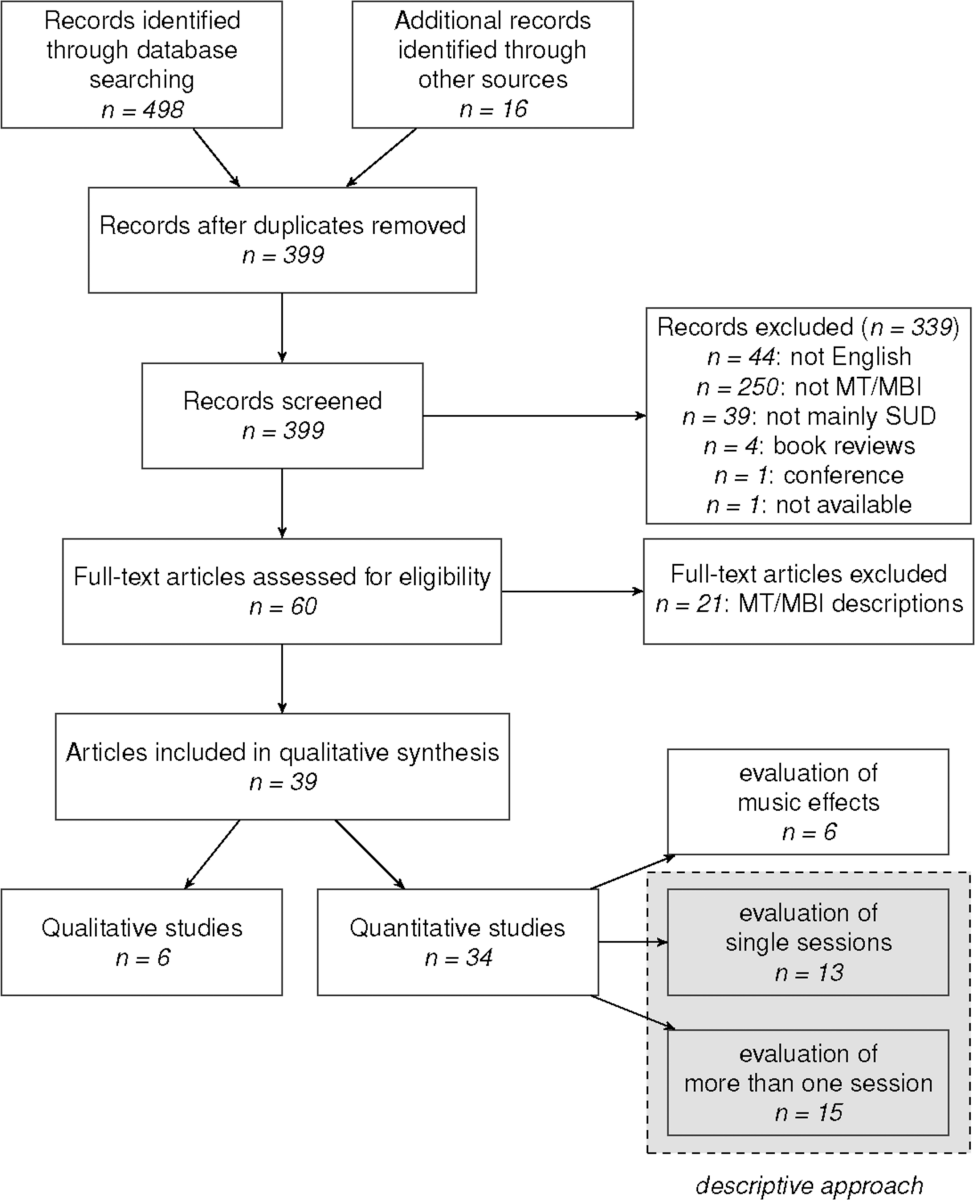
Study inclusion flow chart.

The identification process is displayed in the flow diagram (adapted from the Preferred Reporting Items for Systematic Reviews and Meta-Analyses (PRISMA) guidelines [53]) in Fig 2. Our database search resulted in 383 records (without duplicates), 50 of which met the inclusion criteria. The other records were excluded because (a) they were not written in English (*n* = 44), (b) did not include MT/ MBI as single program or MP (*n* = 250), or (c) did not primarily focus on SUD (*n* = 39). One full-text could not be obtained [54], therefore it was excluded. Five of the initially included records turned out to be book reviews and conference abstracts, thus they were excluded. Full-texts were obtained for the remaining 44 articles and an additional 16 articles were found in their references lists, resulting in a total of 60 records. Twenty-one of them were descriptive articles without structured qualitative or quantitative data and were excluded.

Thirty-nine records with systematic data collection remained. One article included two quantitative studies [20], and one consisted of both qualitative and quantitative studies [55]. Two articles reported about the same dataset [16,56], so that these results were summarized as one study. Altogether, we identified 34 quantitative studies, which were further subdivided with respect to the type of music/ MT that was examined: (1) six studies examined effects of music stimuli presentation without application of MT/ MBI, (2) thirteen studies investigated only one session of MT/ MBI, and (3) fifteen studies examined more than one session of MT/ MBI. Six records included qualitative data obtained through semi-structured interviews, structured or video-taped observations or questionnaires.

### Sample and setting characteristics

The characteristics of the studies are summarized in Table 2 for studies that examined the effects of music stimuli presentation, Tables 3 and 4 for quantitative studies about MT/ MBI, and Table 5 for qualitative studies about MT/ MBI. Sample settings and characteristics are presented separately in the following for (a) MT/ MBI studies with both qualitative and quantitative data, and (b) MP studies.

**Table 2:** Characteristics and results of studies examining effects of music stimuli presentation on patients with substance use disorders.

Study	Outcome	EG	CG	Type of intervention	Frequency/ duration	Measurement tools	Population	Effects
Abdoll-ahnejad[55]	Sleep quality	*N* = 32 m Age: 21–50 polydrug, heroine	-	Listening to relaxing music before bedtime 30 sessions	30 sessions	Questionnaire (time to fall asleep, frequency of nightmares, mood on the following morning, sleep interruptions)	Males Therapeutic community for drug abusers Iran	• Benefits regarding time to fall asleep and mood on the following day ^a^ • Reduced number of visits of the general practitioner ^a^ • Decreased drop-out rate ^a^
Fritz et al.[65]	Positive and negative affect Locus of Control Mood Others	*N* = 22 86.4% m Age: 20–47 (*M* = 32) 78% polydrug	Within subjects JC vs. CC	Musical feedback intervention Music listening to self-produced track (JC) or commercial drum n bass track (CC)	2 sessions (within subjects)	Positive and negative affect scale (PANAS) Internal vs. external locus of control scale Multidimensional Mood Questionnaire (MDMQ) Self-designed items	Rehabilitation program during prison sentence Germany	Effects of condition order: • Internal locus of control higher for JC than CC, when JC firstly presented. * • No mood differences between JC firstly or secondly presented • Increased mood for CC firstly compared to CC secondly presented * • No differences in PANAS • Increased desire to do sports for JC firstly presented vs. JC secondly presented. * Further effects: • People who felt more content, happy, and comfortable thought their training partners were more likeable (*r*_*s*_ = 0.722) and interesting (*r*_*s*_ = 0.702). *** • Increased mood associated with desires to take part in another JC with the same training partner (*r*_*s*_ = 0.774) and to perform activities with the same training partner (*r*_*s*_ = 0.695).*** • Higher mood associated with more internal locus of control after JC(*r*_*s*_ = 0.495).*
Jansma et al.[64]	Desire to drink Mood (distressed, sad, irritated, calm, satisfied) Self-efficacy Physiological measures	*N* = 40 62.5% m Age: 27–59 (*M* = 43.1) 100% alcohol (excluding other drugs)	Within subjects Distressing (high performance task with negative feedback), neutral (reading a magazine), depressing (music)	Mood induction procedure and exposure to an alcohol cue	3 sessions (within subjects)	Visual analogue scales (100mm) Physiological measurement	Inpatient alcohol addiction treatment center Netherlands	Effects on mood: • People receiving depressing MIP were less irritated and more satisfied than those receiving distressing MIP. * • No differences between depressing and distressing MIP for sad and calm. • People were sadder after neutral, distressing and depressing MIP. ** Effects related to desire to drink: • Increase of desire to drink after cue exposure without differences between MIP conditions • Positive correlation between sadness after negative MIP and desire to drink at baseline (*τ* = .26)** Effects related to self-efficacy after cue exposure: • Decreased self-efficacy* without differences between MIP conditions • No correlations with mood after MIP Effects related to physiological measures after cue exposure: • Decreased HR and BP as well as increased HRVm* without differences between MIP conditions • No correlations with mood after MIP
Nerad & Neradová [63]	Music perception	*N* = 45 100% alcohol	Attendants and leaders of training psycho-therapeutic communities *N* = 42	Music listening to major and minor composition	8 sessions, once a week	Questions about chromesthetic music perception	Inpatient antialcoholic treatment Czechia	• EG perceived colors with greater intensity. *** • No differences between major and minor composition • Most frequently perceived colors: yellow for major composition and blue for minor composition (EG and CG)
Short & Dingle[61]	Emotional valence and arousal Craving	*N* = 19 52.6% m Age: *M* = 31.1 95% polydrug 42% alcohol 32% amp 11% cannabis 11% pd 5% heroine	Healthy age- and gender-matched participants *N* = 19	Music listening to 3 stimuli (sad, happy, relaxing songs)	One session	7-point Scale and Geneva Emotions in Music Scale (GEMS-9) single item for craving on 7-point scale, Alcohol urge questionnaire (AUQ)	Residential therapeutic community for SUD Australia	• EG rated happy, sad, and relaxing songs equally. CG rated happy and relaxing songs more pleasant than the sad song. ** Arousal ratings • EG showed no differences in arousal ratings. • CG rated relaxing and sad songs as less arousing than the happy song. * • CG showed higher arousal than EG for happy song $\left(\eta_{p}^{2}=0.21\right)$. ** No differences in arousal ratings between CG and CG for relaxing and sad songs. GEMS-9 ratings • CG had higher intensity of joyful activation for happy song than the EG $\left(\eta_{p}^{2}=0.28\right)$. ** • No differences in GEMS-9 for relaxing or sad song between EG and CG. Craving ratings (CG only) • Effect of time on craving rating for single item $\left(\eta_{p}^{2}=0.51\right)$ and AUQ questionnaire $\left(\eta_{p}^{2}=0.46\right)$.*** • Increase in craving for urge song compared to baseline (single item, AUQ) * • Decrease in craving from urge song to clean song (single item, AUQ) *** • No differences in craving for clean song vs. baseline (single item, AUQ)
Thayer Gaston & Eagle[62]	Music preference LSD experience	Whole sample: *N* = 59 m Age: *Md* = 46.4 100% alcohol Miscellaneous music (EG1): *n* = 16 Familiar music (EG2): *n* = 13 Familiar music with headphone (EG3): *n* = 8 Unfamiliar music (EG4): *n* = 12	No music (*n* = 10)	Music presentation during LSD therapy	One psychedelic session with music presentation under a 500mg dosage of LSD	LSD Music Preference Questionnaire LSD Session Survey Objective Check List for LSD Experience, third party-reports	Males Inpatient alcohol abuse treatment USA	• Changes in the ranking for musical preference for EG3 • Love ballad more preferred across all groups • No differences in LSD Sessions Survey questions between groups • No differences in third-party reports between groups • No reported distortion in the structure of music elected by LSD • Low pitches more noticed than high pitches • Most participants enjoyed the music, statements about the necessity of music during LSD session, most “felt” the music

Studies examining the effects of music/ musical production, not including sessions of music therapy held by therapists or other conducting persons. Effect sizes are only listed when reported in the articles. Amp = amphetamines; BP = blood pressure; CC = Control condition; CG = Control group; EG = experimental group; fm = females; JC = Jymmin’ condition; HR = heart rate; HRVm = heart rate volume; m = males; MIP = mood induction procedure; pd = prescription drugs; SUD = substance use disorders

^a^ Frequency counts

* p < .05.

** p < .01.

*** p < .001

**Table 3:** Characteristics and results of studies examining effects of single music therapy session on patients with substance use disorders.

Study	Outcome	EG	CG	Type of intervention	Frequency/ duration	Measurement tools	Population	Results
Baker et al.[16] Dingle et al.[56]	Perceived enjoyment Engagement Motivation Mood-related experiences	*N* = 24 48.5% m Age: 17–52 (*M* = 34.4) 54% alcohol 30% polydrug/ injecting 13% cannabis	-	MT CBMT (lyric analysis, songwriting, parody, improvisation, singing, listening)	1 session for analysis, 90 min, 7 sessions per week	5-point Likert scale yes-no questions open-ended questions	In- and outpatient rehabilitation unit (detoxification and day patients) Australia	• 75% attendance • 70.8% were at least often motivated to take part in the session • 87.5% mood regulation ^a^ • 65% positive mood change ^a^ • 20% music allowed contact with feelings, relaxing ^a^ • 10% feelings of sadness, depression ^a^ • 83.5% found sessions (extremely) enjoyable ^a^ • 83% would take part in another session • 5.7 ± 2.8 emotions per session; positive: happy, vibrant, comfortable, relieved, inspired, proud; negative: sad, upset, self-conscious, confused ^a^ • Correlation between “use of music to regulate mood” and “help me do something enjoyable without using substances”, *r* = .509 *. • No differences between people with alcohol and drug use disorders for engagement, enjoyment, motivation • No differences between people up to/ over the age of 25 for engagement, enjoyment, motivation
Gardstrom et al.[51]	Anxiety Sadness Anger	*N* = 49 Age: early 20s to late 60s Dually diagnosed with MI and SUD	-	MT composition, listening, improvisation, performance	1 session for analysis 20 sessions, 45min	7-point visual analogue scale	Inpatient dual diagnosis treatment unit USA	• 51% decrease in anxiety, 38.8% no change, 10.2% increase ^a^ • 42.9% decrease in anger, 55.1% no change, 2% increase ^a^ • 65.2% decrease in sadness, 28.6% no change, 6.1% increase ^a^ • 32.7% decrease in all three scales, 20.4% no change in all scales, 0.2% increase in all scales ^a^
Gardstrom & Diestelkamp[19]	Anxiety	*N* = 53 fm *N* = 39 with pre-session anxiety included Alcohol or other drugs, many polydrugs	-	MT composition, listening, improvisation, performance	1 session for analysis 18 sessions, 45min, twice a week, 9 weeks	7-point Likert scale	Females Inpatient gender-specific residential program USA	• 26.4% of the initial sample showed no pre-test anxiety (excluded) ^a^ • 84.6% decrease of anxiety from pre- to posttest ^a^ • 5.1% increase of anxiety ^a^ • 10.3% no change ^a^ • Decrease of anxiety from pre- to posttest ***
Jones[46]	Mood (11 areas) Importance of MT	*N* = 26 (88.5% m) Age: 21–69 (*M* = 39.9) 85% alcohol 58% cocaine 19% cannabis 19% other drugs	Comparison between two MT groups	MT lyric analysis or songwriting	4 days per week	Visual analogue mood scale (100mm) with combined emotions	Inpatient non-medical detoxification facility USA	• Increased feelings of acceptance, joy/happiness/enjoyment ^b^ • Decreased feelings of guilty/regretful/blame, fearful/ distrustful ^b^ • No significant reduction in anxiety/ nervousness/ anticipation, shame/ humiliation/ embarrassment/ disgrace, sadness/ depression, sorrowful/ suffering • No differences between methods • 75% rated MT as significant tool in their recovery (increasing significance with increasing session number). ^a^
Silverman[29]	Motivation (Treatment Eagerness) Working alliance Enjoyment	*N* = 29 Whole sample: *N* = 66 (43.9% m) Age: *M* = 40.8 58% alcohol 12% polydrug 12% pd	Group verbal therapy *N* = 37	MT lyric analysis	1 session 45 min, once a week	SOCRATES (short version) Revised Helping Alliance Questionnaire for therapist and client (HAQ) 7-point Likert scale	Inpatient detoxification unit USA	• No differences in motivation, client-rated working alliance, and perceived enjoyment between EG and CG • Higher therapist-rated working alliance *** for EG vs. CG • All participants noted they would attend another session. ^a^
Silverman[30]	Withdrawal Locus of control	*N* = 64 Whole sample: *N* = 118 (48.3% m) Age: *M* = 40.2	Group verbal therapy *N* = 54	MT lyric analysis	1 session 45 min, once a week	Adjective Rating Scale for Withdrawal (ARSW) Drinking-Related Internal-External Locus of Control Scale (DRIE)	Inpatient detoxification unit USA	• No differences for withdrawal and locus of control between EG and CG • All participants except one noted they would attend another session ^a^
Silverman[31]	Change readiness Depression Enjoyment Helpfulness Comfort Content Being clean	*N* = 69 Whole sample: *N* = 140 (50% m) Age: *M* = 43.2	Group verbal therapy *N* = 71	MT songwriting	1 session 45 min, once a week	University of Rhode Island Change Assessment (URICA) BDI-II 7-point Likert scales and follow-up interview after 1 month Lyric analysis	Inpatient detoxification unit USA	• No differences in change readiness (*η*^2^ = .02) and depression for CG vs. EG • More perceived helpfulness *** (*η*^2^ = .10), enjoyment ***(*η*^2^ = .13), and comfort * (*η*^2^ = .03) for EG vs. CG • No differences in follow-up measures(enjoyment, helpfulness, depression, being clean) between EG and CG (*η*^2^ = .10) • EG more comments regarding enjoyment, thanks, continuation, positive cognitive changes than CG ^a^ • Lyrics concerning consequences of using drugs, insight/change
Silverman[32]	Readiness to change Craving Helpfulness Enjoyment Motivation	*N* = 42 (EG1; Rockumentary MT) *N* = 43 (EG2; Recreational MT) Whole sample: *N* = 141 (58.2% m) Age: *M* = 38.4 55% alcohol 23% heroine 9% pd	Group verbal therapy *N* = 56	MT lyric analysis (EG1) or music bingo (EG2)	1 session 45 min, once a week	Readiness to Change Questionnaire Treatment Version (RTCQ-TV) Brief substance craving scale (BSCS) 7-point Likert scales	Inpatient detoxification unit USA	• RTCQ-TV: Higher scores for Contemplation*** (*η*^2^ = .122) and Action* (*η*^2^ = .052) for EG vs. CG • No differences in craving, helpfulness, enjoyment, and motivation between EG and CG • Correlations between motivation, enjoyment, and helpfulness across all participants** • No differences between EG1 and EG2
Silverman[33]	Motivation and readiness for treatment Content	Posttest *N* = 48 Whole sample: *N* = 99 (48.5% m) Age: *M* = 43.9 64% alcohol 17% heroin 14% pd 3% cocaine	Pretest (wait-list CG) *N* = 51	MT songwriting	1 session 45 min, once a week	Circumstances, Motivation, and Readiness Scales for Substance Abuse Treatment (CMR) Lyric Analysis	Inpatient detoxification unit USA	• Higher scores for motivation * ($\eta_{p}^{2}$ = .068) and readiness for treatment *** ($\eta_{p}^{2}$ = .128) for EG vs. CG • Contents: “action”(*n* = 44), “emotions and feelings”(*n* = 28), “change“ (*n* = 26), “reflection”(*n* = 21), “admission”(*n* = 20), “responsibility” (*n* = 7) ^a^
Silverman[34]	Drug avoidance self-efficacy Motivation for sobriety Treatment eagerness	Posttest *N* = 43 Whole sample: *N* = 131^b^ (53.4% m) Age: *M* = 38.6 57% alcohol 24% pd 17% heroin 2% cocaine	Active CG: group verbal therapy *N* = 41 Wait-list CG: Pretest (with group music bingo) *N* = 47	MT lyric analysis	1 session 45 min, once a week	Drug Avoidance Self-Efficacy Scale (DASES) 7-point Likert scales	Inpatient detoxification unit USA	• No differences for motivation ($\eta_{p}^{2}$ = .001), treatment eagerness ($\eta_{p}^{2}$ = .019), or drug avoidance self-efficacy ($\eta_{p}^{2}$ = .034) between EG and CGs
Silverman[35]	Motivation	Posttest *N* = 49 (EG1 and EG2 with different songs) Whole sample: *N* = 104^c^ (54.8% m) Age: *M* = 41.6 62% alcohol 21% pd 14% heroin 1% cocaine 1% cannabis	Pretest (wait-list CG) *N* = 53	MT lyric analysis	1 session 45 min, once a week	Texas Christian University Treatment Motivation Scale- Client Evaluation of Self at Intake (CESI)	Inpatient detoxification unit USA	• Higher means for problem recognition * ($\eta_{p}^{2}$ = .053), desire for help * ($\eta_{p}^{2}$ = .0.044, treatment readiness **($\eta_{p}^{2}$ = .089), and total motivation ** ($\eta_{p}^{2}$ = .074) for EG vs. CG • No differences between EG1 and EG2
Silverman[36]	Motivation to reach and maintain sobriety Treatment eagerness Knowledge of triggers and coping skills	*N* = 21 Whole sample: *N* = 69 (58% m) Age: *M* = 38.5 58% alcohol 21% heroin 21% pd 1% cocaine	Education without music *N* = 21 Recreational MT (music bingo) *N* = 25	MT educational MT (songwriting)	1 session, 45 min, once a week	7-point Likert scales lists of triggers and coping skills	Inpatient detoxification unit USA	EG with higher motivation than CG1 ** and CG2 ** ($\eta_{p}^{2}$ = .177) • No between-group differences after adjustment for multiple comparisons regarding treatment eagerness, knowledge of triggers and coping skills
Silverman[37]	Withdrawal Current craving	*N* = 60 Whole sample: *N* = 144 (54% m) Age: *M* = 36.8 81% alcohol 42% heroine 10% pd 1% cocaine 1% other	Pretest (wait-list CG) *N* = 84	MT lyric analysis	1 session, 45 min, once a week	Adjective Rating Scale for Withdrawal (ARSW) Brief Substance Craving Scale (BSCS)	Inpatient detoxification unit USA	No differences between the groups regarding withdrawal ($\eta_{p}^{2}$ = .026) or craving ($\eta_{p}^{2}$ = .022). • No relationship between familiarity and withdrawal or craving.

All studies included one session only for data analysis. Effect sizes are only listed when reported in the articles. amp = amphetamines; CBMT = cognitive behavioral music therapy; CG = control group; DARTNA = Drum-Assisted Recovery Therapy for Native Americans; EG = experimental group; fm = females; GIM = Guided Imagery and Music therapy; m = males; MBI = music based intervention; MI = mental illness; MT = music therapy; pd = prescription drugs; SOCRATES = The Stages of Change Readiness and Treatment Eagerness Scale; SUD = substance use disorders

^a^ Frequency counts

^b^ *N* = 121 completed all measures

^c^ *N* = 100 completed all measures

*p < .05.

** p < .01.

*** p < .001

**Table 4:** Characteristics and results of studies examining effects of more than one music therapy/music-based intervention session on patients with substance use disorders.

Study	Outcome	EG	CG	Type of intervention	Frequency/ duration	Measurement tools	Population	Results
Albornoz[39]	Depression (self-rating/ therapist rating)	*N* = 12 Whole sample: *N* = 24 m Age: 16–60 Addiction and depression problem	*N* = 12	MT improvisation (independent therapy)	12 sessions, 2h per week, 3 months	BDI Hamilton Rating Scale for Depression	Males Inpatient treatment for substance abuse Venezuela	• Lower post- than pre-test scores for self-rated depression for EG ** and CG *, *d* = 0.51 across both groups (Power for comparison: 34%) • Lower post- than pre-test scores for therapist-rated depression for EG ** and CG *, *d* = 0.90 across both groups (Power for comparison: 78%) • Lower post-test scores for therapist-rated depression for EG compared to CG *, but not for self-rated depression
Cevasco et al.[43]	Anxiety Anger Depression Stress	*N* = 20 fm Age: 19–42	-	MT competitive games, dancing or rhythm activities	12 sessions, 1h, twice a week each therapy 4 sessions	State-Trait Anxiety Inventory (STAI) Novaco Anger Inventory Short Form (NAI) 10-point Likert scales	Females Outpatient substance abuse program USA	• No overall effects of MT methods, individual effects of MT methods • Average daily percentage of decrease ^a^: Indicated progress for several individuals on decreased levels of depression, stress, anxiety, and anger • Mortality rate: 50%; remaining clients with lower anxiety ** and anger *** values
Dickerson et al.[48]	Treatment retention and completion Substance use Problem severity Comfort and strength derived from spirituality Well-being Cognitive functioning Cultural identity Adoption of principles Physical and psychiatric symptoms	*N* = 10 (50% m) Age: 19–71 (*M* = 52.5)	-	MBI (drumming teacher and counselor) Drum-assisted recovery therapy for Native Americans (DARTNA) (independent therapy)	24 sessions, 3h, twice a week	Substance Use Report Addiction Severity Index, Native American Version (ASI-NAV) Functional Assessment of Chronic Illness Therapy (FACIT): Spiritual Questions Only Expanded, Fatigue (FACIT-F) Functional Assessment of Cancer Therapy–Cognitive Functions (FACT-Cog) American Indian/ Alaska Native Cultural Identity Scale General Alcoholics Anonymous Tools of Recovery (GAATOR 2.1) BSI	Outpatient setting Native Americans USA	• 50% treatment completion (80% until week 6) • Improved psychiatry status * after 6 weeks, improved medical status* after 12 weeks (ASI-NAV) • Spirituality: Improved meaning/peace ** and total score ** after 12 weeks (FACIT) • Improved physical and functional well-being after 12 weeks * (FACIT-F) • No improvements in adoption of principles, physical and psychiatric symptoms or cognitive functions (GAATOR 2.1, BSI, FACT-Cog)
Dougherty[50]	Attendance	Age: adolescent-geriatric 100% alcohol	-	MT music listening (structured sessions) sing along (group)	structured sessions: 3–4 weeks, once a week sing along: 30 min, biweekly	Percent of attendance at any given time	Inpatient rehabilitation/ Therapeutic community for alcohol dependency USA	• 80–90% attendance
Gallagher & Steele[49]	Mood Participation On-task behavior	*N* = 188 Age: 20–59 (*M* = 36) Dually diagnosed with SUD and MI		MT music listening, group participatory music, playing instruments, relaxing, lyric analysis, drumming, songwriting, music and muscle tone/pulse rate	45min, once a week	Roger's (1981) Happy/ Sad Faces Assessment Tool Therapist rating	Outpatient counseling (9 month stay) USA	• 91% active participation ^a^ • 82% expression of thoughts and feelings ^a^ • 68% positive mood changes • 64% no mood changes during the session • 53% not sociable ^a^ • 46% participation in processing the session ^a^ • 60% constricted or blunted affect after the session ^a^
Gallant et al.[44]	Client attitudes Psychosocial functioning	*N* = 6 couples Age: 31–51 (*Md* = 43) Various drug addictions	-	MBI (social worker) music listening, lyric analysis, relaxation	4 sessions, 2h, over 2 weeks	20-Item Hudson Psychosocial Screening Instrument Content analysis	Outpatient recovery Canada	• 5/6 patients rated MT as “very helpful“ • On average clients were less anxious, less depressed, and had fewer relationship problems. • Average Hudson Score decreased from pre to post.* Cohen’s U3 = 88%. • Content: Problem definition (55.8%), problem solving (44.2%), motivation-activation (38%), problem definition (36.6%), assessment (13.1%), goal setting (8.8%), action plan development (3.5%), cognition (68%), affective or emotive expression (32%) ○ “feeling” associated with music (55%) and “thinking” associated with lyrics (78%) more often.***
Howard[42]	Depressiogenic thought frequency State immediate goals	Sample A: *N* = 8 fm Age: *M* = 34.9 Sample B: *N* = 12 adolescents Age: 15–17 Chemical addictions	Within subjects comparison (PT vs. MT) or between samples comparison	MT lyric analysis (PT also including lyric analysis)	6 sessions (alternating music and poetry), 45min, 6 weeks	Automatic Thoughts Questionnaire (ATQ) Goal attainment form (GAF)	2 inpatient substance abuse treatment facilities (rehab-ilitation center) USA	• No differences in depressiogenic thought frequency and state immediate goals between groups or type of therapy for ATQ, GAF, or off-task behavior • High percentage of on-task behavior ^a^
Hwang & Oh[45]	Depression Anxiety Anger Stress	*N* = 42 m^c^ Age: 31–73 (*M* = 50.2) 100% alcohol	Between methods comparison	MT singing, listening, playing instruments (therapist- or patient-selected activities)	12 sessions (4 sessions each therapy) 0.5h, twice a week	10-point Likert Scales	Males Inpatient alcohol treatment program South Korea	• High pretest scores of anxiety, anger, depression, and stress for singing ^a^ • Decreased depression **, anxiety **, anger **, and stress ** in posttest • No differences between methods • No differences between patient- and therapist-selected activities at all • Significant reduction in stress and depression for therapist-chosen activities during singing *
James[20] Study 1	Locus of control	*N = 10* Whole sample: *N* = 20 adolescents (50% m) Age: *M* = 15.8 Chemical addictions	Occupational therapy craft group (waitlist) N = 10	MT music listening lyric analysis	4 sessions, 1h, one week	Abbreviated Internal External Locus of Control Scale	Adolescents Inpatient rehabilitation service for chemical dependency USA	• Greater pre-post increase in internal locus of control for EG than CG *
James[20] Study 2	Locus of control	*N* = 10 Posttest only Whole sample: *N* = 20 adolescents (55% m) Age: *M* = 16.4 Chemical addictions	Occupational therapy craft group (waitlist) Pretest only *N* = 10	MT music listening lyric analysis	4 sessions, 1h, one week	Abbreviated Internal External Locus of Control Scale	Adolescents Inpatient rehabilitation service for chemical dependency USA	• Greater internal locus of control for EG than CG *
K. M. Murphy[38]	Motivation Depression Coping Skills	GIM + standard program *N* = 9 Whole sample: *N* = 16 (56.3% m) Age: 19–55 *M* = 37.2 56.3% polydrug 37.5% alcohol 6.3% cannabis	Standard program *N* = 7	MT GIM (relaxation, imagery focus, music imaging, drawing or journaling)	8 sessions, 50-60min, 21 days	Importance, Confidence, Readiness Ruler (ICR) Beck Depression Inventory (BDI) Sense of Coherence Scale (SOC)	Inpatient residential substance abuse treatment USA	• No differences in coping skills, depression, and motivation between EG and CG in pre- and posttest • Depression ^a^: CG 46% decrease, EG 75% decrease • Retention rate ^a^: CG 50%, EG 75%
Oklan & Henderson[40]	Depression Psychiatric symptoms Coping skills	Case study *N* = 1 m Age: 14	-	MBI (unclear) Recorded Music Expressive Arts (RMEA) therapy with songwriting and production (independent therapy)	16 sessions, 75min, 16 weeks	BDI-II Symptom Checklist 90-R (SC-90-R) Adolescent Coping Orientation to Problems Experienced (A-COPE)	Adolescent Outpatient psychological treatment, inhalant abuse, case study USA	• Depression: Reduced SC-90-R Depression score, no reduction in BDI-II after 10 weeks (normal range) ^b^ • Reduction in obsessive-compulsive, depressive, psychotic, anxiety, and overall symptoms ^c^ • Increased seeking spiritual support, positive imagery, self-reliance ^c^ • Decreased physical diversion, humor ^c^
Ross et al.[15]	Problem Severity General functioning Motivation Physical and psychiatric symptoms Medication adherence Attitudes towards MT and therapist MT characteristics	*N* = 80 (80% m) Age: 20–57 (*M* = 39.7) Dually diagnosed with MI and SUD 50% alcohol 37% cocaine 20% cannabis 19% polydrug 14% opiates	-	MBI (unclear) music and imagery (listening), drumming, improvisation	1h, one to more than 6 sessions	Addiction Severity Index Clinical Global Impression Severity Scale (CGI), Global Assessment of Function Scale (GAF) SOCRATES BSI MT Questionnaire Number of sessions	Inpatient dual diagnosis unit USA	• Pretest variables unrelated to MT characteristics and MT Questionnaire • 100% medication adherence ^a^ • 71% appeared at outpatient aftercare treatment ^a^ • Number of sessions positively associated with aftercare appointment*** Pre- vs. posttest: • Relationship between MT appreciation and changes in CGI ** • Relationship between therapist appreciation and changes in CGI **, GAF *, and Taking Steps * • Relationship between MT appreciation and attitudes towards the therapist *** Cross-sectional analyses at discharge • Relationship between MT appreciation and Taking Steps * Relationship between therapist appreciation and Ambivalence *, Taking Steps*
Silverman[47]	Perceived effectiveness and enjoyment Intervention assessment compared to other groups	*N* = 8 fm Age: 19–65 100% chemical dependency	-	MT music games, relaxation training, lyric analysis, songwriting	8 sessions, once a week	25-point analogue scales	Females Inpatient chemically dependency treatment USA	• No differences between the interventions regarding enjoyment and effectiveness • Mean scores for enjoyment/ effectiveness nearly at maximum ^a^ • 50% reported MT as more effective and enjoyable than other groups ^a^
Yun & Gallant[41]	Forgiveness and grief Depression	*N* = 21 fm Age: 28–64 (*M* = 48) SUD due to forgiveness/grief issues		MBI (counselor) listening, lyric analysis Individual setting	12 sessions per client, 1h, biweekly, 6 month	Forgiveness Grief Perspectives Scale (FGPS) BDI	Females Outpatient rehabilitation center, Canada	• Decrease in forgiveness and grief from pre- to posttest (*d* = 1.95) *** • Decrease in depression from pre- to posttest (*d* = 2.42) *** • Positive correlation between forgiveness/grief and depression in pretest (*r* = .54) ***, and posttest (*r* = .58) ***

Effect sizes are only listed when reported in the articles. For music-based intervention (MBI) studies, conducting persons are listed in brackets. BDI = Beck Depression Inventory; BSI = Brief Symptom Inventory; CG = control group; EG = experimental group; fm = female; m = male; MBI = music-based intervention; MI = Mental illness; MT = music therapy; pd = prescription drugs; SUD = substance use disorders

^a^ Frequency counts.

^b^ Results based on a criterion of clinical significance, i.e., changes by at least one standard deviation of the mean.

^c^ Results based on scores from 36 participants.

*p < .05.

** p < .01.

*** p < .001

**Table 5:** Characteristics and themes of qualitative studies about effects of mt/mbi on patients with substance use disorders.

Study	Type of intervention	Frequency/ duration	Population/ Setting	Measurement tools	Topics/ Themes
Abdollahnejad [55]	MBI (unclear) Lyric analysis, song sharing	25 sessions, 45 min	Therapeutic Community for drug users Iran *N* = 20 m Age: 20–50	• Behavior during the sessions (video tape)	• Increased talking about important issues (e.g., relationships) • Indirect expression of thoughts and feelings • Increased exchange of opinions and experiences • Close interaction between group members (learning about each other, problem solving) • Participants were highly interested • Nostalgic experiences with music related to previous drug abuse
Baker et al.[66]	MT Songwriting	Once a week	Inpatient substance abuse treatment *N* = 5 (40% m) Age: early 20 to middle-aged 60% amp, 40% alcohol	• Reaction during the session • Lyric analysis	• Incidental rebellion • Lengthy process of group problem solving, personal reflection, reevaluation • Clear engagement (declined smoking break) • Safe medium for the expression of negative emotions • Humor
Eagle[60]	MT Listening to music (during LSD therapy)	5 times per day, 30 min each,	Inpatient alcohol abuse treatment USA *N* = 16 m Age: 34–59 100% alcohol	• Behavioral observations with therapist’s notes (structured case studies)	• Importance of familiar music • Important contents: Religion and love • Nonverbal communication through music between patients and therapist • “Music “guides” patients’ experiences through the LSD therapy sessions.” (p. 35)
Liebowitz et al.[57]	MBI (vocal performance majors) Choral music program	Once a week, 75 min Quarterly performance	Residential facility for homeless veterans with SUD Southwestern USA *N* = 6 (66.7% m)	• Individual semi-structured interviews ○ Duration of the association with the study site ○ Duration of the participation in the choir ○ How learned about choir ○ Expectations ○ Experiences ○ Interaction with the context ○ Impact on relationships ○ What they would tell other veterans about the choir	• Personal motivations ○ Opportunities to meet other residents ○ Affinity to singing ○ Diversion their attention from other contents ○ Opportunity to learn (singing, music) ○ Personal challenge • Emotions linked to participation ○ Anxiety ○ Enjoyment ○ Elevating effect on mood, relaxing • Perceived intragroup dynamics ○ Belonging, commitment to the choir ○ Support, enhanced performance
Rio[59]	MT Improvisational music	Once a week, 2h 10 months data collection	Church-based shelter with Choirhouse church choir USA *N* = 3 m consistent members Age: 26, 45, 55 (*M* = 42) 66% polydrug 33% cocaine	• Behavior during the sessions (video tape, session notes, personal journal, audio tapes) • Individual semi-structured interviews in the first month ○ History, interest in music ○ Feelings, thoughts ○ Relationships ○ Music ○ Substance abuse ○ Medical, mental health issues	• Consistent attendance and intense involvement of the core group members • Identified themes: ○ Emotional expression (grief and loss, joy, state of being) ○ Beauty and spirituality (aesthetic, character, faith, altered states) ○ Relationships (support, closeness, difficulty, connecting) ○ Story (history, metaphor, shared experiences) ○ Structure (boundaries, traits, music) ○ Create/Risk (making music, void) ○ Health (psychological, physical/cognitive)
Zanker & Glatt [58]	MBI (artists of Council for music) Music listening	Twice a week, 30 min	Inpatient mental hospital UK Alcoholics and narcotics	• Questionnaires about individual attitude towards music and mood after listening	• Diversity and subjectivity of reactions to music • Expression of emotions through music • Group cohesion dependent on personality • Reactions to music can reflect personality aspects • Congruity between mood states and intrinsic character of music linked to improvement of clinical status and long-term outcomes • Music may serve as diagnostic tool (projection of mood into music)

For music-based intervention (MBI) studies, persons conducting the sessions are listed in brackets. MBI = music- based interventions; MT = music therapy.

#### MT/ MBI studies

For the majority of the studies, sessions were held in group settings, except a single-case study [40] and one study with individual application of the music-based program [41]. Most of the studies, i.e., three qualitative and 23 quantitative studies, were classified as “MT studies” (according to the music therapy definition provided in Introduction). With respect to MBIs, one study was conducted by vocal performance majors [57], one by different artists of the Council of Music [58], one by a cultural drumming teacher and a substance abuse counselor [48], one by a social worker [44], one by a counselor [41], and in three cases [15,40,55] the therapist’s background remained unclear.

Not considering the case study, sample size ranged from 8 participants [42,47] to 188 participants [49] for the quantitative studies, and from 3 participants [59] to 20 participants [55] for the qualitative studies. One quantitative [50] and one qualitative study [58] did not report sample sizes.

Six studies examined men only [40,45,50,55,59,60] and five women only [19,41–43,47].

Regarding the diagnosis, many samples included various drug addictions, i.e., polydrug abuse. Other studies only focused on chemical dependency [20,42,47], alcohol [45,50,60] or inhalant abuse [40].

With respect to the age of the participants, four studies investigated adolescents only with mean ages/ age ranges between 15 and 17 years [20,42] or as a single case study with a 14-year old boy [40]. For the other studies, mean age varied from 34.4 years [16] up to 52.5 years [48]. Eleven studies [16,19,39,43,47,50,51,55,57,58,60] did not report any measure of central tendency regarding age. In 16 cases [15,16,38,39,41,43–49,55,59,60] numeric age ranges were reported which varied from 21 years [44] (31–51 years) to 53 years [48] (19–71 years).

#### Music stimuli presentation studies

Sample sizes ranged from 19 participants [61] to 59 participants [62].

Two studies examined men only [55,62], and three investigated both men and women. One study did not report any information about gender [63].

Regarding the diagnosis, three studies focused on alcohol addiction [62–64], and the others included various drug addictions.

Regarding the age, mean age ranged from 31.1 years [61] to 43.1 years [64]. Two studies did not report any measures of central tendency [55,63] and one reported a median age of 46.4 years [62]. Age ranges (when reported) differed only slightly from 28 years [65] (20–47) to 33 years [64] (27–59).

### Results of quantitative MT/ MBI studies separated by outcomes

**Fig 3:**
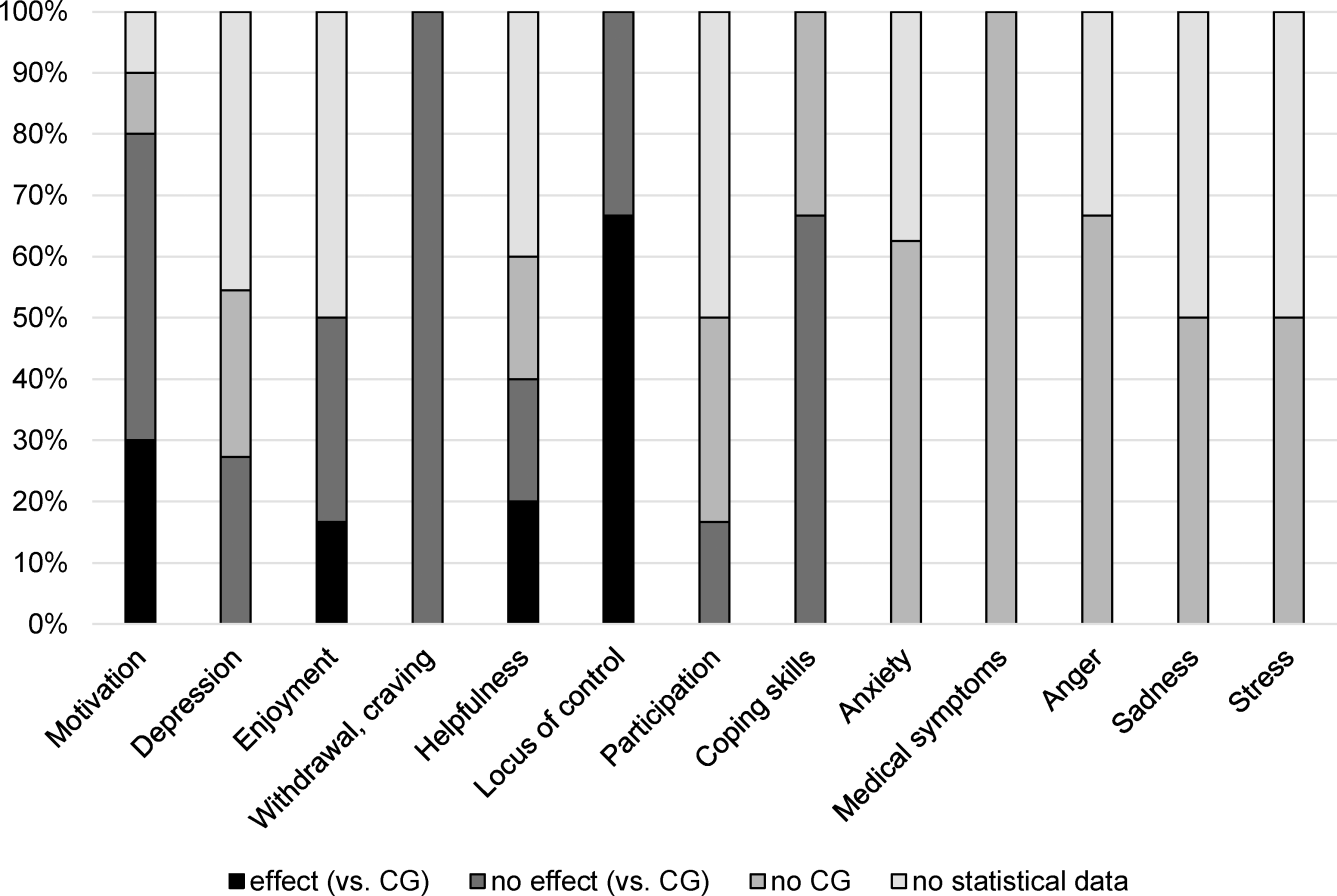
Descriptive overview of quantitative studies examining the effects of music therapy and music-based interventions on different outcomes. Studies with effect or no effect compared to control group (CG) were classified as of high level evidence of efficacy (black and dark grey bars). Studies reporting insufficient statistical data to conduct meta-analyses and without CG were classified as of low level evidence of efficacy (light grey bars).

For an overview of the efficacy of MT/ MBI per outcome (cluster) in consideration of the quality of the studies see Fig 3. Studies were classified according to the categorization scheme presented in Fig 1. None of the studies met the criteria of categories (3a) and (3b), i.e., studies with CG without randomization, so that these categories are not represented in Fig 3. In the following section, we will describe the results in more detail.

#### Motivation

For the effect of MT/ MBI on variables related to this cluster (motivation, treatment eagerness, change readiness), 10 results were collected, and eight of them (80%) represent high level evidence of efficacy. For Silverman [32,34] who examined different motivational constructs within the same samples only motivation scores were used. All studies except one reported all statistical data and only one included pilot data without a CG [15]. In 37.5% of studies of high level evidence of efficacy (3/8), i.e. 30% of all studies (3/10), beneficial effects of MT/ MBI were found. All RCTs except one [38] were conducted by Silverman [29,31–36], and they differed widely with respect to CG designs and scales, so a meta-analysis was not conducted.

#### Depression

This outcome was examined in 11 studies including Howard [42] which reported on two separate samples. Five studies report insufficient statistical data and four were non-controlled studies, so that only 27% of the results (3/11) were categorized as of high level evidence of efficacy. None of the RCTs found benefits of MT/ MBI compared to CG. A meta-analysis was not conducted due to the different CG designs.

#### Enjoyment

All three data sources of high level evidence of efficacy (i.e., 50% of all studies regarding this outcome) were reported by Silverman [29,31,32], so that we did not conduct a meta-analysis. Three further studies of low level evidence of efficacy [16,46,47] did not report all necessary statistical data. One out of three RCTs, i.e., 17% of all results (1/6), reported a positive effect of MT on enjoyment.

#### Withdrawal and craving

We decided to cluster these outcomes as the variables are closely linked. Patients in states of withdrawal often experience craving, and consumption of the substance may immediately and effectively reduce the symptoms [37]. Silverman [30,32,37] conducted three different RCTs examining craving and/or withdrawal in patients addicted to various drugs (e.g., alcohol, heroine, prescription drugs and cocaine). None of the studies showed beneficial effects of MT compared to different CG. A meta-analysis was not conducted because all results were reported by the same author.

#### MT helpfulness

Forty percent of the results (2/5) were of high level evidence of efficacy, comparing MT to group verbal therapy, and both RCTs were conducted by Silverman [31,32], so a meta-analysis was not appropriate. The lack of statistical details prohibited inclusion in meta-analysis for two further studies [44,47], and another study was a non-controlled study [15]. All in all, 50% of studies of high level evidence of efficacy, i.e., 20% of all studies (1/5) regarding helpfulness were in favor of MT/ MBI.

#### Locus of control (LOC)

All three studies considering LOC were RCTs, and two of them [20] (i.e., 67%) found positive effects of MBI/ MT. A meta-analysis was not conducted due to different CG designs.

#### Participation

For this category, many different constructs regarding the quantitative assessment of patients’ engagement and participation were subsumed, so six data sources were identified: Only one study included a CG [29] and did not identify benefits of MT. Furthermore, 50% of all data sources (3/6) did not report all statistical data [16,49,50] and 33% (2/6) were non-controlled studies [15,48].

#### Coping skills

Only one study out of three studies (33%) for this outcome, was of low level evidence of efficacy, i.e., a case study not including a CG [40]. None of the RCTs [36,38] found benefits of MT for coping skills.

#### Constructs examined without studies of high level evidence of efficacy

For five outcome clusters, namely *anxiety*, *medical symptoms*, *anger*, *sadness*, and *stress*, no RCTs could be identified, so conclusions about efficacy cannot be drawn.

#### Follow-up investigations

Only one RCT assessed follow-up scores regarding depression, enjoyment, perceived effectiveness and being clean [31] and did not identify differences between group verbal therapy and MT groups one month after intervention completion.

#### Conclusion

For at least eight categories of outcomes, studies of high level evidence of efficacy, i.e., RCTs, were identified. The descriptive summaries suggest that there is evidence for benefits of MT/ MBI compared to different control groups (CGs), especially for the variable locus of control (67% positive effects compared to CG). Additionally, regarding perceived helpfulness of the intervention, half of the RCTs reported higher values for MT compared to CG. For motivation and enjoyment there were inconsistent results, and more than half of the studies of high level evidence of efficacy did not identify statistically significant improvement for MT/ MBI participants. Regarding depression, withdrawal/ craving, participation, and coping skills none of the RCTs reported benefits for MT. Studies examining anxiety, medical symptoms, anger, sadness, and stress were all of low level evidence of efficacy, so that their results can only serve as a base for further research giving hints to constructs that should be evaluated with RCTs.

### Results of quantitative studies separated by study characteristics

We now describe effects of MP, MT and MBI considering study characteristics according to the following categories: (1) effects of music in general, (2) effects of one session of MT/ MBI, and (3) effects of more than one session of MT/ MBI. Because most of the studies were not RCTs, conclusions about MT efficacy cannot be drawn. Thus, the summaries include descriptions of clinical effectiveness, i.e. the effects in clinical practice [67].

Studies comparing MT methods (e.g., lyric analysis and songwriting [46]) did not identify significant differences between the interventions, so that the methods are not differentiated in the following. With respect to the nomenclature, we noticed that regarding mood there is still no consensus, as *mood*, *feelings*, and *emotions* are often used interchangeably. For instance, Jones [46] refers to the terms “feelings and emotions” (p. 100), only to eventually assess “mood” using a visual analogue mood scale. Thus, due to the heterogeneity of the nomenclature used in the studies, it was not possible to differentiate these terms properly.

#### Effects of music presentation (MP)

Six studies examined the impact of music on patients with SUD without therapeutical involvement of an interventionist (see Table 2). The following effects of listening to music were reported: Short and Dingle [61] examined the impact of sad, happy, and relaxing songs on arousal in patients with SUD and a healthy control group (CG). Whereas the participants of the CG indicated different degrees of arousal and pleasantness for the three tracks, the SUD patients rated the three pieces of classical music equally arousing and pleasant. Furthermore, their degree of craving was linked to the personal relevance of songs: The patients reported increased craving after listening to a track associated with their substance use, whereas afterwards, listening to a track associated with abstinence resulted in decreased craving. These results indicate less emotional variations in SUD patients and a direct impact of music on relapse related variables. Furthermore, Fritz et al. [65] reported a strong context dependency of music effects. They conducted a musical feedback intervention with listening to a jointly self-produced music piece or a commercial track. Self-produced music showed positive effects on mood and locus of control (LOC) only when it was presented prior to the commercial music production. Jansma et al. [64] examined the effect of mood states on alcohol cue reactivity. They induced depressive mood by presentation of depressive music or distressed mood by negative feedback following a high performance task. Alcohol cue reactivity was present, but did not differ between negative or neutral mood states. Nevertheless, the patients were less irritated and more satisfied after depressive mood induction compared to distressing mood induction.

With respect to more abstract outcomes, patients with alcohol dependency perceived colors with greater intensity after listening to music compared to people (patients and staff of a therapeutic community) without exposure to music [63]. Similarly, music during LSD therapy was associated with colors, geometric designs, and past events. Between groups with and without music exposure, there was no difference in LSD experience. Nevertheless, only listening to familiar music appeared to have an effect on general music preference [62].

Additionally, there was experimental evidence for positive effects of music listening over a longer period of time. For members of a therapeutic community for drug users, music listening before falling asleep was related to increased sleep quality and mood on the following day as well as to decreased drop-out rate during a one-month-intervention [55].

#### Effects of one MT/ MBI session

MT/ MBIs typically include more methods than simply listening to music [68]. Effects of quantitative studies examining single MT sessions (mostly lyric analysis, songwriting or improvisation) are summarized in Table 3. Most of them were conducted in detoxification centers with a short duration of stay between three and five days. Compared to a verbal therapy CG, MT participants showed similar measures of change readiness, depression, sobriety [31], client-rated working alliance [29], LOC [30], treatment eagerness, drug avoidance self-efficacy [34], craving [32], and withdrawal symptoms [30]. Silverman compared MT groups to wait-list CGs with pretest only, and found no differences regarding craving and withdrawal [37]. Positive effects of MT vs. group verbal therapy were found for therapist-rated working alliance [29], comfort [31], and motivational variables: MT participants had higher realization that aspects of change can be better than the status quo and more active changes [32]. In line with that, MT groups showed increased problem recognition, desire for help, treatment readiness, and total motivation compared to a wait-list CG with pretest only [33,35]. Furthermore, Silverman [36] found higher motivation to reach and maintain sobriety for participants of educational MT compared to patients receiving education without music or a music game. In the same study, treatment eagerness and knowledge of coping skills or triggers did not differ between groups. In three other studies, similar motivation scores between MT groups and verbal therapy or pretest CG were identified [29,32,34], indicating that the effects of single MT sessions on motivational aspects are not coherent. Regarding perceived enjoyment and helpfulness, the results were not consistent as well [29,31,32].

Other studies with single sessions for data analysis were conducted in an inpatient non-medical detoxification unit [46], an in- and outpatient rehabilitation unit [16], an inpatient dual diagnosis treatment unit [51] and an inpatient gender-specific residential program [19]. All these studies reported beneficial effects on mood: For instance, 65% of the participants showed a positive mood change [16]. More specifically, a great amount of the participants reported decreased anxiety [19], anger, and sadness [51], and or an increase in acceptance, enjoyment, happiness, and joy [46]. Furthermore, 87.5% of the participants used MT for mood regulation [16]. Nevertheless, one study found no differences between pre- and posttest regarding anxiety and depression [46].

#### Effects of multiple MT/ MBI sessions on mood

Effects of studies examining more than one session are summarized in Table 4. Awareness, expression, and change of emotions are often mentioned as important intended therapy goals [50]. Therefore, five studies in inpatient settings [38,39,42,45,47] and five studies in outpatient settings [40,41,43,44,49] examined treatment effects on mood and emotions. Generally, MT participation was associated with positive mood changes [49], and the scores for perceived enjoyment and effectiveness of MT were almost at the maximum [47]. With respect to negative emotions, MT was linked to reduced anger, depression, stress, and anxiety [40,41,43–45]. Two RCTs identified beneficial effects of MT regarding therapist-reported, but not self-reported depression scores [38,39].

#### Effects of multiple MT/ MBI sessions on other outcomes

MT and MBI also affected other psychological variables: Adolescents with chemical dependency completing MT showed increased internal LOC compared to a wait-list CG engaging in alternative activities [20]. Results regarding motivation and coping skills were not clear: While in one RCT similar levels for both variables after standard treatment (CG) and additional Guided Imagery and Music (GIM) therapy were reported [38], a single case study found improved coping skills and motivation [40]. This patient had also reduced psychiatric symptoms after the MT intervention. In line with this finding, a cultural-based drumming treatment was associated with improved psychiatric and medical status in Native Americans [48]. In a non-randomized pilot study conducted in an inpatient treatment for dually diagnosed people with SUD and mental illness, Ross et al. [15] examined relationships between MT variables, psychiatric symptoms, general functioning, aftercare appointment, and motivation measured by the Stages of Change, Readiness and Treatment Eagerness Scale (SOCRATES). They found positive associations between MT appreciation and global functioning during hospital stay. Therapist appreciation was positively related to changes in global functioning and the Taking Steps subscale of SOCRATES measuring active changes. Furthermore, cross-sectional analyses at discharge revealed associations between MT appreciation and Taking Steps as well as between therapist appreciation and the Ambivalence and Taking Steps subscales of SOCRATES. Although MT variables did not directly predict improvement in psychiatric symptoms, the number of attended sessions was positively related to aftercare appointment in a following outpatient program within one week after hospital discharge. With regard to long-term effects beyond the hospital treatment, MT was also associated with sobriety and reduced substance use in another study [48]. As this pilot sample consisted of a small number of Native Americans not involved in inpatient settings, it remains unclear whether the results are transferrable to other populations. Nevertheless, MT was associated with beneficial behavioral aspects like high involvement, attendance and on-task behavior in several studies [42,49,50]. These findings suggest that MT and MBI may be important tools for recovery in line with the participants’ subjective evaluations of treatment effects and perceived helpfulness.

### Qualitative studies

Six qualitative studies examined and described the participants’ reactions, attitudes, and subjective associations in the context of MT and MBI. In four studies, the patients’ behavior during the session was recorded using video-tapes [55,59] therapist’s notes [60,66] and lyric analysis [66]. Some authors conducted semi-structured interviews [57,59] or used questionnaires that were analyzed qualitatively [58]. Four general themes were identified: Firstly, music served as a tool for expression of thoughts and feelings. Secondly, in all qualitative studies the role of music and MT/ MBI for group interaction, cohesion, and relationships to others, including the therapist [60] was emphasized. Thirdly, MT/ MBI were related to the learning of skills regarding music [57], problem solving [66], and social interaction [59]. Finally, MT/ MBIs were associated with benefits for health and quality of life [59]. In line with the quantitative data, the behavioral observations revealed high engagement and involvement of the participants [55,59,66].

## Discussion

In order to address the research question whether music therapy (MT) and music-based interventions (MBIs) are clinically efficient for people with substance use disorders (SUD), we obtained a systematic collection of articles resulting in 34 quantitative and six qualitative studies. Regarding MT/MBI efficacy, we used a descriptive approach to summarize the efficacy evidence of quantitative studies. Furthermore, we summarized effects of exposure to music stimuli, MT and MBIs to describe findings regarding effectiveness. In the following, we discuss these effects, focusing on motivation and on findings regarding the four main themes identified in qualitative analyses. Furthermore, we discuss the quality of the studies. Taken together, the studies do not show clear common effects. Additionally, only few studies have assessed outcomes related to substance use even though such outcomes are critical for treatment success. Thus, variables such as long-term sobriety need to be examined in future studies. Possible mechanisms that may contribute to positive effects of MT/MBI remain to be investigated and specified as well.

### Effects of music stimuli presentation

There is evidence for the direct impact of listening to music on emotions and craving without application of MT/ MBI [61]. In addition, frequent listening to relaxing tracks had a beneficial effect on sleep, mood, and treatment completion [55]. Neuro-imaging studies have demonstrated that music listening engages many brain structures important for cognitive, emotional, and sensorimotor processing [69], in particular the mesocorticolimbic system [70,71]. Positive short-term effects on variables like craving may reflect benefits for mental health even on a neurobiological level [72].

### Short-term effects of single MT/ MBI sessions

Apart from the general impact of music stimuli presentation, participation in single MT sessions may result in additional short-term effects. Those are important to examine because many patients with SUD attend detoxification treatments with a low frequency of therapy sessions [3]. Single MT sessions appear to be as effective as single verbal therapy sessions for various psychological outcomes (e.g., withdrawal, LOC, craving, client-rated working alliance, and depression), and there were higher scores for MT for comfort [31], therapist-rated working alliance [29], and some aspects of change readiness [32]. These findings support the use of MT in short-term treatments for SUD. Results regarding enjoyment, helpfulness, and motivation differed between studies [29,31,32], although these aspects may be especially important in short-time interventions. As they may be related to positive therapeutic experiences, these factors may facilitate the participation in additional interventions. Importantly, the only RCT with follow-up assessment did not find any beneficial effects of single MT sessions on depression, enjoyment, perceived effectiveness and sobriety [31] after a one-month period. Additional longitudinal analyses of single session effects are necessary.

### Effects of MT/ MBI on motivation

Lack of motivation is a crucial problem in the treatment of SUD [73], and beneficial effects of MT and MBI on motivation were commonly described [74,75]. Music itself is motivating and empowering for many people and it has been suggested that engagement in music making may lead to enhanced internal change motivation [76]. High rates for on-task behavior and engagement reported in qualitative and quantitative studies included in this review support this assumption [55]. Ten studies quantitatively assessed motivation, and eight of them were RCTs. Most of them investigated single sessions [29–36], and two included longer interventions [15,38]. Despite the positive qualitative reports of patients, not all of these studies identified significant benefits for MT/ MBI. Silverman reported higher treatment and sobriety motivation after MT compared to a wait-list CG with pretest only [33,35], whereas others identified no differences compared to verbal therapy or pretest [32,34]. Different results may be due to different study designs, comparisons or measurement instruments. For instance, a Likert scale for the assessment of motivation revealed similar ratings across groups, whereas the use of a multidimensional scale resulted in higher scores for experimental group than CG in the same sample [32]. In line with that, most studies with Likert scales did not identify benefits for MT groups [32,34,36], whereas the use of some multidimensional instruments revealed significant treatment effects [32,33,35]. There is actually no consistent definition for motivation in the context of research on addiction [77]. Therefore, it is difficult to find an adequate outcome measure capturing all relevant aspects and fitting to the treatment setting. For instance, Silverman [33] examined treatment motivation and readiness with the Circumstances, Motivation, and Readiness Scales for Substance Abuse Treatment [78] and did not identify benefits for MT. The use of this instrument as a clinical assessment tool is not recommended [77] because it was originally developed in the context of a therapeutic community. It is, at this point, not possible to claim that issues with instrument selection are related to incongruence of findings; however, this is certainly an issue worthy of further investigation in future studies.

Prochaska and DiClemente [79] argued that behavior change always occurs as process with different stages of change, so that differentiating aspects of motivation regarding these stages might be useful. Considering this, beneficial effects of MT on problem recognition, desire for help, treatment readiness, and overall motivation were reported [35]. Furthermore, there might be a benefit of therapeutic use of music compared to solely music engagement without therapeutic context as MT participants showed higher motivation scores than patients playing a music game instead [36]. Nevertheless, there were no differences for treatment eagerness in the same study, suggesting that there is need to differentiate between the motivational variables. More RCTs that use the same outcome measures and use the same control group interventions are needed to draw further conclusions.

Examining more than one session of MT, K. M. Murphy [38] did not identify benefits in motivation for patients with an additional GIM intervention compared to those with standard program only. Because this study did not include a sufficient amount of participants (*N* = 16), long-term effects on motivation should be systematically examined in larger samples in more detail.

### Effects of MT/ MBI on mood and emotions

In many studies, MT/ MBI had beneficial effects on mood and emotions, i.e., positive mood change, decreased negative emotions, e.g., anxiety, depression, and anger, and increased positive feelings, e.g., enjoyment and happiness. This is in line with the importance of MT for the expression and regulation of feelings, as identified in our qualitative analyses. MT provides opportunities for the exploration and expression of feelings without drugs and appears to be a non-threatening intervention [80]. Therapist-selected songs as well as songs written or selected by the participants themselves contain aspects related to feelings [33]. Many music therapy studies have demonstrated that songs may be used as a verbal and nonverbal tool for the exploration of feelings [12,16,46,81,82]. Jones reported that over the course of lyric analysis and songwriting interventions, emotional expression appears to increase, and suggests that positive mood changes may have a positive influence on further treatment-related variables such as therapeutic alliance [46]. Additionally, support by other group members may facilitate emotional expression [50]. Nevertheless, it should be noted that for many emotional variables (e.g., anxiety, anger, sadness) RCTs are needed to assess MT/ MBI efficacy.

### Effects of MT/ MBI on skills and locus of control

Qualitative analyses suggested that MT/ MBI provide opportunities to learn in various areas. Many patients with SUD have poor psychosocial skills, which improved over the course of MT/ MBI [46,59]. M. Murphy [83] has suggested that music, as part of the participants’ everyday life, is adaptable to low levels of psychosocial functioning, and group interventions may be helpful in reducing social isolation. According to Ghetti [76], in group music therapy sessions, the therapist structures the active music making purposefully to enable group interaction in a non-threatening atmosphere. Successful group interactions in music making may help to develop social and problem solving skills. Furthermore, discussion of lyrics of popular songs can help enhance understanding of the individual’s dynamics regarding substance use and may lead to the development of more healthy coping strategies [76]. Only very few studies examined effects of MT/ MBI on cognitive abilities quantitatively. In contrast to the findings reported in qualitative studies, in RCTs no positive effects of MT on coping abilities could be identified [36,38], and also no effects of MBI on cognitive functioning were reported [48]. In contrast to that, a single case study showed enhanced coping after individual sessions [40]. However, these studies differed with respect to many variables, e.g., age, drugs, MT/ MBI methods, and duration so conclusions regarding treatment effect cannot be drawn at this time.

As internal change motivation is a critical aspect for the treatment of addictions, effects of MT/ MBI on locus of control (LOC) were examined as well. After a single session, MT participants did not differ regarding LOC compared to a verbal therapy group [30]. Furthermore, in an experimental setting examining effects of music stimuli presentation, increased internal LOC depended on the context [65], but after longer MT interventions, enhanced internal LOC was identified [20]. These results suggest that MT/ MBI may lead to increased internal LOC over time. When patients experience that their own abilities and actions determine what happens [84] during MT/ MBIs, this may be transferred to life outside the therapy setting and result in better outcomes of addictions’ therapy in the long term [85]. Typically in MT, music experiences are carefully structured and supported by the music therapist to enhance the potential for positive experiences by the patient [86]. This may lead to positive effects of MT on factors such as self-esteem [87] or self-efficacy [88]. However, it is important to acknowledge that asking patients to engage in music making may lead to some anxiety and insecurity as well for some patients, as has been reported in studies outside of the SUD population [89]. However, no studies to date have directly examined the relationship between mastery in music therapy and long-term treatment outcomes for patients with SUD. More research is necessary to explore this possible mechanism.

### MT/ MBI effects on group interaction and relationships

Positive group dynamics were identified as important motivators in all qualitative studies. Over the course of the intervention, behavioral observations revealed increased exchange and cohesion [57,59,66]. Nevertheless, in their study with offenders in a substance abuse/mental illness treatment program, Gallagher and Steele [49] reported that 53% of their participants were “not sociable” (p. 121). For planning of the sessions, clinicians need to keep in mind that many patients with SUD have poor social skills. However, none of the quantitative studies in our review systematically examined group-related variables, so future research should examine social skills or aspects like group cohesion. Summarizing studies with respect to the outcome cluster, participation reveals a lack of studies of high level evidence of efficacy regarding this topic as well.

Regarding working alliance between therapist and patients, beneficial effects from the therapist’s perspective were identified quantitatively [29] as well as qualitatively [60]. By contrast, patients attending MT did not perceive a better working alliance compared to a verbal therapy CG [29]. This is in line with previous studies identifying weak reliability between therapist-rated and patient-rated working alliance in drug treatment [90]. Regarding the relationship between different perspectives of working alliance and therapeutic success, results are not consistent: Some studies found stronger relationships between the counsellor’s/ therapist’s view and success [90–94], whereas in other studies the patient’s view was identified as a more important predictor [95] or both measures were only weakly correlated with success [96]. Furthermore, levels of working alliance had different effects on outcome for different types of therapies [97]. These inconsistent results indicate that working alliance may be more complex and depend on many aspects. As most of the studies emphasized the importance of the therapist’s view, especially ratings at early time points after starting the therapy [98] as examined by Silverman [29], working alliance should be examined in further MT studies.

### MT/ MBI effects on quality of life and health

In many studies, MT and MBI were associated with a great amount of perceived enjoyment and also reported to enhance quality of life and improve health [59]. In line with this, longer MBI were related to positive psychiatric and medical outcomes [40,48]. Nevertheless, these investigations were conducted in very specific settings, so that there is still a lack of studies examining health-related and long-term variables in common treatments for SUD. Especially, variables related to substance use are understudied. Furthermore, all studies examining medical symptoms were categorized as of low level evidence of efficacy in our descriptive summaries. Thus, high quality evidence has not been conducted.

### Study quality and methodological recommendations

Our descriptive summaries considered the quality of the identified studies and revealed that in the last years, since the review of Mays et al. in 2008, more RCTs were conducted. Thus, for outcomes like motivation, depression, enjoyment, withdrawal and craving, perceived helpfulness, working alliance, and locus of control studies of high level evidence of efficacy already exist. Nevertheless, we did not calculate meta-analyses due to study heterogeneity or because similar variables were only examined by the same author. Furthermore, across all studies included in our descriptive approach, still only 38% (25/65) were RCTs, and especially for mood variables and long-term abstinence, high quality research has not been conducted. Due to the low quality of most of the studies, in the end, strong key outcomes cannot be substantiated.

It is important to consider that in studies that examine the impact of group interventions, the independence of observations, a common assumption for standard statistical tests, may have been violated because of interactions between group members. This may have resulted in biased standard errors and erroneous inference [99].

In Table 6, methodological recommendations are summarized that are aimed at helping to overcome issues in future research. Most importantly, studies should investigate long-term outcomes such as abstinence and use randomized controlled trial designs. In order to reduce problems related to the independence of observations, hierarchical analyses taking into account the group structure of the data or cluster randomization should be applied. However, designing and executing of cluster randomized trials is difficult because for example larger sample sizes are needed or recruitment bias could occur [100].

**Table 6:** Methodological recommendations summary.

• Inclusion of long-term outcome variables such as abstinence and attendance of aftercare treatment programs
• Hierarchical data analysis
• Studies with randomized-controlled trial designs, and if randomization is not possible in the clinical context at least inclusion of a control group
• For all types of studies reports about characteristics of the interventions, studies and participation with transparent information about statistical procedures
• Reports of standardized effect sizes
• Inclusion of outcome variables related to skills (e.g., cognitive abilities), group dynamics and relationships (e.g., group cohesion, working alliance), and life quality and health (e.g., medical symptoms, general functioning)
• Use of standardized measurement instruments suitable for addiction and music therapy contexts
• Inclusion of external researchers who are not interventionists

If in the clinical context randomization is not possible, studies should at least include control groups as reference frameworks. In within subjects designs aimed at examining pre to post MT/MBI intervention improvements in functioning, one needs to consider that the statistical regression to the mean may be an explanation for the patients’ improvement. Including a control group may solve this issue. Studies of low level evidence of efficacy can be useful for generating hypotheses, getting information about subjective experiences, exploring effects on individual levels, or assessing the ecological validity of treatments [25]. Thus, we also included them in our review, but in 50% of these non-RCT studies (20/40), the results were reported without sufficient statistical information. Furthermore, across all studies, reports about characteristics of intervention, studies and participants varied widely, so that giving a transparent overview and comparing the studies regarding these aspects was difficult. In addition to that, only few studies reported standardized effect sizes [31–37,39,41], so the effects of MT/ MBI could hardly be interpreted and compared across studies. Therefore, we recommend the inclusion of reports that clearly describe characteristics of intervention, studies and participants, including diagnostic criteria, transparent information about statistical procedures, and all necessary statistical data (including effect sizes) according to the guidelines of the Task Force on Statistical Inference [101] in the articles. In addition to that, as described in the paragraphs above, high-quality research for outcomes related to skills, group interaction and relationships has not been conducted although these aspects are important topics mentioned in qualitative research. Thus, future studies should investigate variables such as cognitive abilities, group cohesion or medical symptoms among others. Measurement instruments for the same outcomes widely varied across studies (e.g., Likert scales vs. standardized tests) and they mostly captured different aspects, so comparisons were difficult. Therefore, in future research authors should use the same standardized measurement instruments that are suitable for the addiction and music therapy context. Furthermore, the researcher often acted also as music therapist and collected the data which may lead to procedural bias (such as Rosenthal effect [102]) or response bias in the data. It also remains unclear whether effects are due to the music therapy or the person of the music therapist. To reduce these tendencies, we recommend the inclusion of external researchers for data collection and analysis.

## Conclusions

There is still no consensus regarding the effects of music therapy (MT) and music-based interventions (MBI) for patients with substance use disorders (SUD). Previous reviews [21,22] highlighted the need for more randomized controlled trials (RCTs) regarding long-term outcomes like maintenance of sobriety. The current literature includes additional RCTs, but most of them focused on short-term effects after single sessions in detoxification units. One RCT examined sobriety after a one-month period without significant differences between a single session of MT or group verbal therapy [31]. The only study examining abstinence after more than one session was conducted with one specific ethnic group without inpatient participants [48]. Therefore, future studies should include long-term investigations and follow-up measurements, in particular regarding variables related to substance use. Due to the great fluctuation in SUD treatments, planning of these studies may be a challenge. However, reduction of substance use and abstinence are critical aspects regarding the success of addictions’ treatment, so evaluations of treatment effects for these outcomes are necessary for future investigations. MT/ MBI appeared to be effective in the regulation of emotions and subjective outcomes, as also indicated by qualitative analyses. Nevertheless, the quantitative studies in our review were very diverse which was one important reason for not conducting meta-analyses. As MT/ MBI are commonly and specifically used in the treatment of groups and subgroups with SUD, e.g., women or adolescents [8], it is important to examine its efficacy and effectiveness in these specific populations as well. However, these results may not be generalizable across general SUD settings. Additionally, it is important to be aware that music can also trigger relapse (e.g. if the music is associated with substance abuse [61]), and that, therefore, music has to be used with great care in SUD patients.

Regarding limitations of the current review it must be noted that collecting the characteristics of the studies was particularly difficult because of missing information. We did not consider the patients’ additional diagnoses and treatment options, methods or specific therapy goals. These topics could be included in future reviews to provide additional insights in characteristics of effective MT/ MBI/ MP. Due to the small number of MT studies, separations regarding these aspects are currently not useful. Whereas this systematic review summarizes the available evidence in terms of treatment efficacy, it does not provide information about potential mechanisms of action of MT/ MBI for SUD. Furthermore, a methodological review of MT/ MBI/ MP and SUD studies may be warranted in the future. For example, studies could be codified regarding methodological strengths and weaknesses to make further methodological recommendations with respect to the investigation of concrete outcomes.

From a methodological point of view, future studies examining the efficacy of MT/ MBI/ MP for patients with SUD should include RCTs, so that meta-analytic calculations will be possible. Regarding content and outcome variables, future studies should consider including outcomes related to the qualitative findings as well as variables related to substance abuse so that a comprehensive picture of the efficacy of MT/ MBI/ MP can be drawn. In addition, we urgently need mechanistic studies that identify and examine the impact of potential treatment mediators and moderators. Additionally, the effects on problem solving, cognitive, and coping abilities and the role of MT/ MBI/ MP for different stages of motivation should be clarified. Furthermore, effects of the interventions on long-term medical and psychiatric outcomes, treatment retention and completion should be examined, while considering additional moderating and mediating variables like MT appreciation. Based on these findings, implications for future MT/ MBI as independent or adjunctive treatment programs for SUD can be developed. As individual preferences regarding music and MT as well as group dynamics appeared to be important for the success of MT [21], careful group composition and selection of materials are necessary. All in all, due to its high acceptance, flexibility, easy accessibility and low costs, MT/ MBI provide opportunities for SUD treatment for various groups in various settings. Nevertheless, its efficacy and effectiveness have to be evaluated more systematically and should focus on further long-term outcomes.

## Supporting information

S1 Table Prisma 2009 checklist for the review about music therapy and music-based interventions in the treatment of substance use disorders. (PDF)
